# The impact of interventions for youth experiencing homelessness on housing, mental health, substance use, and family cohesion: a systematic review

**DOI:** 10.1186/s12889-019-7856-0

**Published:** 2019-11-14

**Authors:** Jean Zhuo Wang, Sebastian Mott, Olivia Magwood, Christine Mathew, Andrew Mclellan, Victoire Kpade, Priya Gaba, Nicole Kozloff, Kevin Pottie, Anne Andermann

**Affiliations:** 1 University of Ottawa Faculty of Medicine, Bruyere Research Institute, University of Ottawa, Ottawa, ON Canada; 2 McGill University Faculty of Medicine, Montreal, QC Canada; 3 C.T. Lamont Primary Health Care Research Centre, Bruyere Research Institute, Ottawa, ON Canada; 4 Bruyere Research Institute, Ottawa, ON Canada; 5 University of Toronto, Faculty of Nursing, Toronto, ON Canada; 6 Department of Family Medicine, University of Ottawa, Ottawa, ON Canada; 7 Centre for Addiction and Mental Health, Department of Psychiatry and Institute of Health Policy, Management and Evaluation, University of Toronto, Toronto, ON Canada; 8 Departments of Family Medicine and Epidemiology and Community Medicine, Bruyere Research Institute, University of Ottawa, Ottawa, ON Canada; 9 Department of Family Medicine and Department of Epidemiology, Biostatistics and Occupational Health, Faculty of Medicine, McGill University, Montreal, QC Canada

**Keywords:** Youth, Homelessness, Vulnerably housed, Interventions, Gender, Equity

## Abstract

**Background:**

Youth often experience unique pathways into homelessness, such as family conflict, child abuse and neglect. Most research has focused on adult homeless populations, yet youth have specific needs that require adapted interventions. This review aims to synthesize evidence on interventions for youth and assess their impacts on health, social, and equity outcomes.

**Methods:**

We systematically searched Medline, Embase, PsycINFO, and other databases from inception until February 9, 2018 for systematic reviews and randomized controlled trials on youth interventions conducted in high income countries. We screened title and abstract and full text for inclusion, and data extraction were completed in duplicate, following the PRISMA-E (equity) review approach.

**Results:**

Our search identified 11,936 records. Four systematic reviews and 18 articles on randomized controlled trials met the inclusion criteria. Many studies reported on interventions including individual and family therapies, skill-building, case management, and structural interventions. Cognitive behavioural therapy led to improvements in depression and substance use, and studies of three family-based therapies reported decreases in substance use. Housing first, a structural intervention, led to improvements in housing stability. Many interventions showed inconsistent results compared to services as usual or other interventions, but often led to improvements over time in both the intervention and comparison group. The equity analysis showed that equity variables were inconsistently measured, but there was data to suggest differential outcomes based upon gender and ethnicity.

**Conclusions:**

This review identified a variety of interventions for youth experiencing homelessness. Promising interventions include cognitive behavioural therapy for addressing depression, family-based therapy for substance use outcomes, and housing programs for housing stability. Youth pathways are often unique and thus prevention and treatment may benefit from a tailored and flexible approach.

## Background

Youth homelessness is a major public health challenge worldwide, even in high income countries [1]. Youth experiencing homelessness are defined as, “youth between the ages of 13 to 24 who live independently of their parents or guardians, but do not have the means to acquire stable, safe or consistent residence, or the immediate prospect of it [2].” Youth pathways into homelessness are anomalous and seldom experienced as a single isolated event. Compared to the adult homeless population, youth experiencing homelessness are more likely to report leaving home due to parental conflicts, including: being “kicked out” of the home, abuse (physical, verbal, sexual and other), parental neglect due to mental health problems, or parental substance use [3–11]. The broader context of family dysfunction can lead to youth circumstances that further reinforce situations of homelessness, including desire for separation from unsupportive environments, financial independence, mental health challenges, substance use, and/or run-ins with the justice system [1].

Not only are youth’s pathways into homelessness different from the adult homeless population, but their experiences on the street are distinct as well. Once homeless, youth are exposed to many dangers and are at a high risk of further trauma [12]. Youth experiencing homelessness may face a number of daily stressors and have limited coping strategies and resources to deal with these stressors [13]. Youth homelessness is often invisible and includes vulnerable housing situations such as couchsurfing or staying with relatives [14]. Furthermore, youth experiencing homelessness are vulnerable to social and health inequities, which describe the fairness in the distribution of health opportunities and outcomes across populations [15]. Health inequities are differences in health status that are unfair and/or avoidable [16]. Often, the compounding effect of various stratifying characteristics can result in increased disparities between individuals.

Current research has largely focused on adult populations, with a gap in evidence on interventions for youth experiencing homelessness on a broad range of outcomes. Among the current interventions for individuals experiencing homelessness, non-abstinence contingent permanent supportive housing and case management have shown promising results in terms of improving housing stability and mental health outcomes [17]. However, youth are a distinct population and they require specifically tailored, context appropriate, equity-focused interventions and research attention [18]. From systematically searching the literature for youth interventions, this paper will introduce four main categories of interventions applied to youth experiencing homelessness: 1) individual and family therapies (ie. cognitive behavioural therapy, motivational interviewing, etc.) 2) skill building programs, 3) case management, and 4) structural interventions (such as housing support, drop-in centres, and shelters). These interventions are designed to address the complex, multifaceted pathways and contributors to youth homelessness, whether it be addressing substance use issues through motivational interviewing, mental health care through cognitive behavioural therapy, improving unstable family environments through family therapies, increasing access to resources through case management, and enhancing structural support such as income and housing support [19–23]. Given the complexity and interconnectedness of these outcomes, one would hope that these interventions would have an impact on not only the primary outcome, but also extend to other facets of a youth’s life. For instance, family therapies have shown promising results on both family functioning as well as substance use, by addressing the toxic family environment and thereby decreasing its contribution to unhealthy substance use patterns [24].

Current research on interventions for the population of youth experiencing homelessness lacks a comprehensive synthesis on a broad range of social and health outcomes. The objective of this review is to synthesize the existing scientific literature on interventions for homeless or vulnerably housed youth in high income countries, and assess the impacts of the interventions on housing, mental health, substance use, and family cohesion, with an equity perspective.

## Methods

We established an expert working group consisting of homeless health researchers, academics, clinicians and youth with lived experience of homelessness to conduct this review. We report our results according to PRISMA-E [see Additional file 3] and published an open access protocol on the Campbell and Cochrane Equity Methods website [25, 26].

### Data sources and search strategy

Without language restrictions, we systematically searched the following databases from inception until February 9, 2018: Medline, Embase, CINAHL, PsycINFO, Epistemonikos, HTA database, NHSEED, DARE, and Cochrane Central. Combinations of relevant keywords and MeSH terms were searched, including “homeless” and “homeless youth” [see Additional file 1 for search strategy]. We hand-searched included studies for primary studies and consulted experts for additional papers. We conducted a grey literature search on homeless health and public health websites.

### Inclusion and exclusion criteria

We downloaded citation information into Rayyan online software [27]. All title and abstracts were screened according to our inclusion criteria (see Table 1) in duplicate by two independent reviewers, and any discrepancies were resolved. Throughout a process of several consultations, our working group, consisting of persons with lived experience and experts in the field, helped develop these inclusion criteria by identifying priority areas in which to focus this review. This study focused on youth between the ages of 13 to 24, however, the age categorizations of youth tend to differ between various definitions, with the medicolegal definition utilizing ages 16 to 21. It is important to note that the broader age range utilized in this paper may lead to risks of over-inclusion, but it was chosen as it is reflective of the currently literature on youth homelessness and includes both high school and university students who are generally still dependents living with family or relying on them for financial or moral support.

**Table 1:** Eligibility criteria

Study Characteristics	Inclusion Criteria	Definitions
Population	Youth between the ages of 13 to 24 who live independently of their parents or guardians, but do not have the means to acquire stable, safe or consistent residence, or the immediate prospect of it [2]. This age range was chosen as it is reflective of the current literature on youth homelessness and includes both high school and university students who are generally still dependents living with family or relying on them for financial or moral support. Furthermore, this definition of homelessness accounts for hidden homeless youth who may not be found in institutional settings but may be couch-surfing with friends or others.	
Interventions	Youth Interventions	Youth interventions are intended to assist youth experiencing homelessness in improving health or social outcomes, which includes both interventions that are created specifically and solely for the benefit of youth as well as interventions for all persons that are applied to the context and needs of youth. Interventions include any program, service, structure, or resource provided with the aim of addressing social and health outcomes. Examples of youth interventions include, but are not limited to, cognitive behavioural therapies and family-based therapies. Cognitive behavioural therapy takes into account emotional, familial and peer influences to build self-control, self-efficacy and reduce negative behaviours [28]. Family-based therapy focuses on intrapersonal factors and re-establishing connections; it seeks to understand individual behaviour and interactions between the individual and their family [20, 29]. Parental monitoring intervention programs providing parenting skills and empowering parents of adolescents [30]. Street outreach and addictions services consist of outreach workers engaging youth living on the street to enhance their wellbeing through programs such as mobile harm reduction programs [31].
Comparison	Any study with a comparison intervention was included, such as standard intervention, alternative intervention, or treatment as usual.	
Outcomes	Studies were not excluded based upon the reported outcomes	
Study Characteristics	Randomized control trials and systematic reviews. All study designs must include interventions with a comparison/control group and have measured outcomes.	
Study Characteristics	Exclusion Criteria	Justifications
	Studies taking place in low- middle-income countries Studies that exclusively report on Indigenous specific interventions	Due to the variability in access to resources and supports in comparison to that in a high-income country, we feel that the settings are different and should be synthesized separately. The analysis of the interventions tailored to this population will be covered by a separate research group.

### Data extraction and analysis

Data extraction proceeded in duplicate using a standardized data extraction form and a third reviewer resolved discrepancies [25]. We extracted data regarding the effectiveness of interventions on a broad range of social and health outcomes. We conducted a scoping exercise to identify key outcome categories in the literature and prioritized reported outcomes with our expert working group members, which included individuals of lived experience. The outcomes rated as being of highest priority (mental health, substance use, housing, and family outcomes) are reported in the body of this paper, and the remaining outcomes (violence, sexual health, personal and social, and health and social service utilization) are reported in the appendix [see Additional file 2]. To reduce overlap between single studies and systematic reviews, we reported the results of systematic reviews and supplemented with data from randomized control trials (RCTs) that were not included in the systematic reviews. Due to heterogeneity of interventions and outcomes studied, we qualitatively synthesized the results. We created a forest plot to summarize RCTs for mental health outcomes, as sufficient data were available and it was a highly ranked outcome.

### Health equity analysis

We used the PROGRESS+ framework to apply a health equity lens and enable us to identify characteristics that socially stratify youth experiencing homelessness, and various drivers of homelessness [15]. In particular, we extracted the following from studies to inform our analysis: 1) study rationale for focusing on youth-centred interventions; 2) the measures used to assess differences in outcomes for women and men; 3) the study’s gender-related findings and conclusions; and 4) the study’s incorporation of equity considerations (e.g. race/ethnicity and socioeconomic status).

### Critical appraisal

We assessed the methodological quality of systematic reviews with AMSTAR II and RCTs using the Cochrane Risk of Bias Tool [28–32]. When assessing the overall risk of bias of RCTs, we defined the risk of bias as “not serious” when there were low risk ratings in all categories or one or two unclear risk, “serious” with one or two high risk categories, and “very serious” with more than two high risk categories.

## Results

**Fig. 1:**
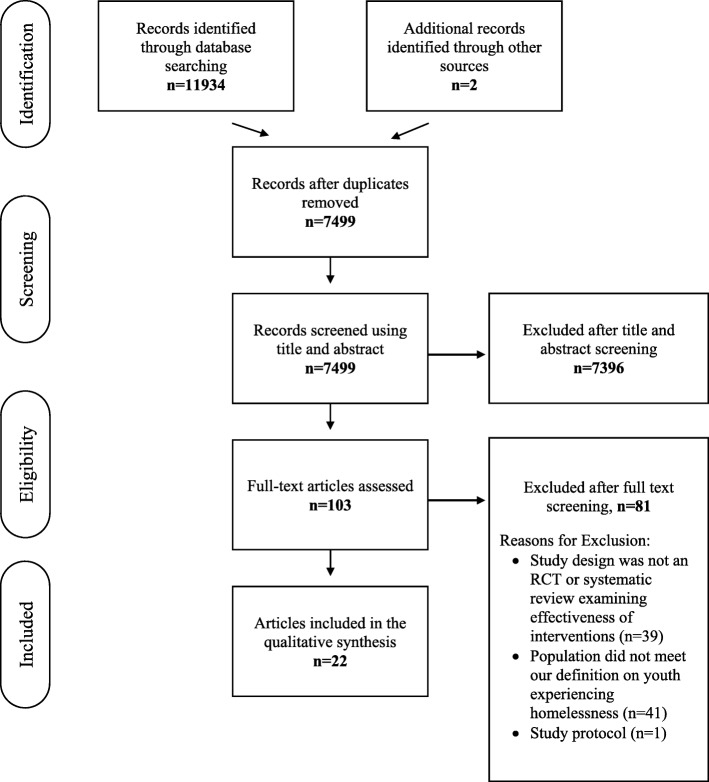
PRISMA Flow Diagram

The search strategy yielded 11,934 potentially relevant citations. After we removed duplicates, we screened 7499 citations and assessed 103 full text articles. Twenty-two citations met the full inclusion criteria (See Fig. 1). Four of the included citations were systematic reviews [33–36] and the remaining 18 citations reported on 15 RCTs (see Table 2 for RCTs and Table 3 for SRs) [19, 21, 37–53].

**Table 2:** Characteristics of Included RCTs

Study	Population	Sample Size (n)	Setting & Country	Intervention	Control	Outcome Measures and Follow-Up Time Intervals (Follow up rates)	Conclusions of the paper
Baer 2007	Youth, ages 13 to 19, vulnerably housed Mean age 17.9 Males 56%- Females 44% Ethnicity was reported as 58% Caucasian, 19% multiracial, 9% Native American, 8% African American, 4% Hispanic or Latino, and 2% Asian or Pacific Islander.	*n* = 117	Community Drop in Center- USA	Brief Motivational Intervention; up to 4 sessions; Average session length was 17 mins for 1st and 35 mins for 2nd session (*n* = 66)	Service as Usual (*n* = 51)	1. Substance use 2. Service use 3. Counsellor ratings of engagement 4. Treatment exposure and satisfaction Measurements were conducted at 1 month (82.9%) and 3 months (76.1%) post intervention.	The purpose of this study was to build upon previous mixed findings. However, the Brief Motivational Intervention did not lead to any improved outcomes in youth compared to those in the treatment as usual group.
Bender 2015	Youth, ages 18–21, Mean age 19 homeless 68.9%- housed 31.1% Males 60.8%- Female 36.5%- Other 2.7% Ethnicity was reported as White 41.9%- Black 20.3%- Latino 5.4%- other 32.4%	*n* = 97	Youth homeless Shelter -USA	SAFE (Safety Awareness for Empowerment); 3 day group intervention of 6–8 youth; focus areas include mindfulness, skill-building (*n* = 56)	Shelter services as usual, which includes case management services (*n* = 41)	1. Mindfulness scores (total, observing, describing, acting with awareness, accepting without judgement) Measurements completed as posttest at the end the intervention. The control group youth completed the interview approximately 5 to 7 days after their baseline interview (F/U for control 90.2% and for intervention 94.9%)	The SAFE intervention led to a significant increase in mindfulness, defined as observation skills, compared to those receiving services as usual. This suggests that youth experiencing homelessness are likely to engage in mindfulness training in shelters.
Greeson 2015/ Courtney 2008	youth age 17; in out of home care- Males 41.15%- Females 58.85%- Ethnicity was reported as White 8.97%- Black 40.17%- Hispanic 43.38%- Other 7.48%	*n* = 482	Independent living programs for youth in foster care- USA	Life skills training course (LST); two 3 h sessions per week for 5 weeks at community college (*n* = 234)	Services as usual aimed at preparing youth at risk of aging out of foster care (*n* = 248)	1. Interventions and service use 2. Job preparedness and preparedness 3. Education and employment 4. Economic well-being 5. Housing 6. Delinquency 7. Pregnancy 8. Documentation and accounts 9. Social support Measured over three time points (baseline, 1 year (91%), and 2 years (88%))	The in-class life skills training course did not appear to be more effective than services as usual to improve social support and other reported outcomes in youth. More research is required to determine the types of youth based interventions that lead to an improvement of the desired outcomes.
Guo 2016 / Slesnick 2013a / Slesnick 2013b)	youth 12–17 years; met DSM-IV criteria for alcohol/drug abuse; Mean age 15.4 Males 47.5%- Female 52.5% Ethnicity reported as White non- Hispanic 25.7%- African American 65.9%- Hispanic 1.7%- Native American 1.1%- Asian American 0.6%- Other 5%	*n* = 179	Short term Crisis Center for Run-away adolescents- USA	1. Community Reinforcement Approach (CRA) - 12 sessions, operant conditioning approach to teach methods of addressing life problems without alcohol or drugs (*n* = 61) 2. Ecologically-based Family Therapy (EFT) - 12 sessions, works with youth and family to identify dysfunctional interactions and improve social interactions (*n* = 57) 3. Motivational Enhancement Therapy (MET) - 2 therapy sessions, improve intrinsic motivation to change drug and alcohol use (*n* = 61)	Three arm study, see interventions	1. Family cohesion and conflict 2. Internalizing behaviours and externalizing behaviours 3. Substance use Measurements were conducted at baseline and at 3, 6, 9, 12, 18, and 24 months post intervention (F/U rates ranged from 69 to 79% across 6 time points and did not differ statistically between groups)	Ecologically-based Family Therapy is more effective and has longer lasting effects on family dynamics compared to individual therapies. While there are many challenges in the implementation of family-based therapies, overcoming these barriers will lead to improved family outcomes in youth.
Hyun 2005	male youth; residence in shelter; mean age 15.5; without psychiatric disorders- 100% male- 0% female Ethnicity not specified	*n* = 27	Shelter for runaway and homeless youth-South Korea	Cognitive behavioural therapy - 8 sessions over 8 weeks (*n* = 14)	Service as usual, no cognitive behavioural therapy (*n* = 13)	1. Self-esteem and self-efficacy 2. Depression Measurement interval was a Pretest- posttest design (F/U rates 87.5% for experimental group and 81.2% for control group)	This study shows that CBT is a useful intervention to increase self-efficacy and decrease depression in youth compared to no treated. These results are in agreement with previous studies showing the effectiveness of CBT in youth to improve mental health outcomes.
Kozloff 2016	youth aged 18–24; homeless or vulnerably housed; with mental disorder Mean age 21.5 Gender reported as Non- male 39% Ethnicity was reported as White 38% -Ethnoracial 36%- Aboriginal 26%	*n* = 156	Participants were recruited from community agencies that serve homeless people, institutions, including health care facilities and prisons, and directly from the street- Canada	Housing first with Assertive Community Treatment or Intensive Case Management (*n* = 87)	Service as usual (*n* = 69)	1. Housing stability 2. Quality of life 3. Employment Measured at baseline, 6, 12,18 and 24 months post intervention (89.7% F/U for the intervention and 72.3% for the control)	Housing First significantly improved housing stability in homeless youth with mental illness compared to those in the treatment as usual group. This is an effective intervention to improve the stability of homeless youth and reduce the long-term negative outcomes of this population. However, since the intervention did not have a significant effect on the other measured outcomes, it is recommended to adjust the intervention to better meet the needs of youth.
Krabbenborg 2017	Homeless youth aged 17 to 26 Average age 20 68.1% male- 31.9 female Ethnicity reported as 51% had a Dutch background	*n* = 251	Shelters for Homeless young adults- Netherlands	Houvast: A strengths-based intervention focusing on improving quality-of-life of homeless youth (*n* = 134)	Services as usual, such as housing, social network, education and finances (n = 117)	1. Mental and physical health 2. Quality of life 3. Violence 4. Income security 5. Satisfaction with family relations 6. Substance use 7. Autonomy 8. Competence 9. Resilience Measured in two waves at baseline as youth enter shelter and as the youth existed the homeless shelter, between 27 and 238 days – mean 156 days post baseline. (F/U 77.6% for control and 80.3% for intervention group)	Both the strength-based intervention and care as usual improve outcomes of homeless youth. No significant differences were found between the two groups. This suggests that youth benefit from receiving care services in general.
Milburn 2012	Families with youth ages 12 to 17; vulnerably housed; no current abuse or neglect Mean age 15.6 Males 33.8%- Females 66.2% Ethnicity reported as White 11.3%- African American 20.5%- Hispanic 61.6%- Other Mixed 6.6%	*n* = 151	Community based organizations- USA	STRIVE: 5 weekly home-based sessions focused on family conflict resolution and problem solving (*n* = 68)	Standard care received from the agencies that referred them (*n* = 83)	1. Substance use 2. Delinquent behaviour 3. Risky sexual behaviours Measured at baseline, 3 (71%), 6 (58%), and 12 months (46%) post intervention	Youth receiving the STRIVE intervention had a significantly decreased number of sexual partners and decreased usage of substances, excluding marijuana, compared to those receiving standard care. Youth receiving the intervention may have increased their marijuana use to replace alcohol and hard drugs.
Peterson 2006	youth; 14–19 years; vulnerably housed; recent binge drinking episode without recent alcohol or drug treatment Mean age 17.4 Males 54.7%- Female 45.3% Ethnicity reported as Caucasian 72.3%- African American 3.2%- Native American 3.2% -Hispanic/Latino 3.2% mixed race 15.9%- Asian/Pacific Islander or other race less than 1%	*n* = 285	Street or community agencies -USA	Brief Motivational Intervention: 1 session lasting on average 30 mins, provide information about patterns and risks (*n* = 92)	2 control groups: 1. Assessment only (*n* = 99) 2. Assessment at follow up only (*n* = 94)	1. Alcohol and drug use 2. Stage of change for substance use Measured at baseline, 1 month (82%) and 3 months (80%) post intervention	The Brief Motivational Intervention led to a decrease in illicit drug use, apart from marijuana, after one month of follow-up compared to those in the control group. Other results of the study were inconclusive and future research should focus on how and when desired outcomes are achieved.
Slesnick 2016	youth; 14–24 years; recent alcohol use; homeless; did not receive drop in, mental health, substance use services in past 3 months Mean age 20.8 Male 53.2% Female46.8% Ethnicity reported as White, not of Hispanic origin 57.0% Other 43.0%	*n* = 79	Drop in Center and Shelter- USA	1. 6 months of strengths-based outreach approach linked with drop-in center (n = 40)	1. 6 months of strengths-based outreachapproach linked with crisis center (*n* = 39)	1. Contact with services 2. Alcohol use 3. Personal control/self-efficacy 4. Depressive symptoms 5. Health (physical and mental) Measured at baseline 3,6,9 months post intervention (3,6,9 month F/U rates were 87,87,90% for the shelter linkage and 88,90,93% for the drop-in linkage conditions, respectively)	This study showed that the drop-in center intervention was more effective to link youth to services and led to an overall increase in service usage than the crisis center intervention. Youth in both groups reported an improvement in mental health and substance use outcomes, with no significant difference between the groups. However, youth in the intervention group demonstrated a reduction in drinking to the point of intoxication.
Slesnick 2015	youth; 14–20 years, vulnerably housed; met DSM IV criteria for abuse or substance disorder Mean age 18.74 Males 52.59% Females 47.41% Ethnicity reported as White non Hispanic 19.6% African American 65.56% Hispanic 2.22% Native American 0.74% Asian American 0.37% Other 11.48%	*n* = 270	Drop in Center- USA	1. Community reinforcement approach provided through a drop-in center 2. Motivational enhancement technique – two 1 h sessions through a drop-in center 3. Case management - 12 1 h sessions through a drop-in center	Three arm study, see interventions	1. Substance use 2. Depressive symptoms 3. Internalizing and externalizing problems 4. Coping 5. Victimization during the last 3 months 6. Homelessness (12 months) Measured at baseline 3,6, and 12 months post intervention (F/U 58.1% for CRA, 88.4% for MET, and 63.7% for case management)	Youth receiving the community reinforcement approach had improved substance use outcomes compared to those in the other two groups. However, while youth in all three arms had an improvement in the other reported outcomes, there was no significant difference between groups.
Slesnick 2009	Youth; 12–17 years; primary alcohol problem; family reside within 60 miles of research site; parents must have agreed to the possibility of family therapy. Mean age 15.1 years males 45% females 55% Ethnicity reported as African American 5% Anglo 29% Hispanic 44% Native American 11% Other 11%	*N* = 119	Runaway shelters- USA	1. Home-based ecologically based family therapy (EBFT) (*n* = 37), 2. Office-based functional family therapy (FFT) (*n* = 40)	Service as usual case management through a drop-in centre (*n* = 42)	1. Substance use 2. Psychological functioning 3. Family functioning Measured at baseline, 3 (75%), 9 (76%),15 (76%) months follow up post intervention. There were no statistically significant differences between groups in attrition	Youth in all three groups showed improvement in substance use, psychological functioning and family functioning. Family therapy has a greater impact on decreasing days of substance use compared to service as usual. Mixed results were obtained in the comparison of home-based family therapy compared to office-based. Therefore, more research is necessary to identify the most effective context of family therapy.
Slesnick 2007	youth; 14–22 years; vulnerably housed; met DSM-IV criteria for Alcohol or other Psychoactive Substance Use Disorders Mean age 19.21 Males 66% Females 34% Ethnicity reported as Native American 13% Asian 1% African American 3%, Hispanic 30% Anglo 41%, and mixed ethnicity/race 12%	*n* = 180	Drop in Center- USA	Community Reinforcement Approach: 16 treatment sessions offered, average 6.8 per participant (*n* = 96)	Service as usual through the drop-in center. The center offered a place to rest, food, showers, clothing and case management (*n* = 84)	1. Substance use 2. Mental Health (Individual functioning, depression) 3. Social stability Measured at baseline and at 6 months post intervention. (F/U 84% for CRA, and 88% for control)	Youth who received the community reinforcement approach had statistically significant improvements in mental health and substance use outcomes compared to those receiving treatment as usual. While youth in the control group also demonstrated improvements in certain areas, the effects of the intervention were more significant and long-lasting since it aimed to improve the relationship between homeless youth and their environments.
Thompson 2017	youth; 17–22 years; engaged in unprotected sex or heavy drinking Mean age 19.3 Females 58.3% Ethnicity reported as Hispanic 47.5% African American 36.1% Other race/ethnicity 16.4%	n = 61	Crisis center- USA	Two session individual brief intervention (45–60 min): focused on changing alcohol and HIV risk behavior (*n* = 30)	Two session educational comparison (*n* = 31)	1. Alcohol use 2. HIV sexual risk behaviours 3. Alcohol related sexual risk 4. Readiness to change alcohol use 5. Readiness to change HIV sexual risk behaviors 6. HIV preventive knowledge Measured at baseline and at 1 month (87.1%) post intervention	The brief intervention did not improve alcohol use outcomes in youth compared to those in the educational comparison group. However, it did improve the willingness of youth to change their alcohol behaviour. Future research is necessary to demonstrate how to translate willingness to change behaviour to an actual change in behaviour.
Tucker 2017	youth 18–25 years Mean age 21.81 Male 73% Female 27% Ethnicity reported as 31% non-Hispanic white 31% African American 25% Hispanic 24% multiracial/other 21%	*n* = 200	Drop in Centers-USA	AWARE: 16 weekly 45-min sessions of group motivational interviewing (*n* = 100)	Service as usual which includes access to food, hygiene services, case management and other programs available at the drop-in center (n = 100)	1. Alcohol, marijuana and drug use 2. Sex related outcomes Measured at baseline and 3 months post interventions. (95% F/U for intervention and 86% for control)	Youth in the AWARE group had decreased frequency in alcohol use and unprotected sex compared to those in the treatment as usual group. While there was an improvement of willingness to reduce marijuana and other drug use, there were no improvements in the frequency of use. This may be because the intervention did not make specific references to marijuana or other drugs.

**Table 3:** Characteristics of Included Systematic Reviews

Study	Design and Quality	Objective	Included Studies	Population	Interventions	Results/Outcomes
Altena 2010	Systematic Review AMSTAR 5/13 Critically low quality review	“To provide a summary of effective interventions for homeless youth by collecting, summarizing, categorizing, and evaluating quantitative studies.”	n = 11 Cauce (1998) [RCT, 150, USA] Slesnick (2007) [RCT, 180, USA] Peterson (2006) [RCT, 285, USA] Baer (2007) [RCT, 117, USA] Hyun (2005) [RCT, 32, South Korea] Upshur (1985) [Quasi-experimental, 57, USA] Upshur (1986) [Quasi-experimental, 22, USA] Ferguson (2008) [Quasi-experimental, 28, USA] Fors (1995) [Quasi-experimental, 221, USA] Kisely (2008) [Quasi-experimental, 45, Canada] Slesnick (2008) [Uncontrolled pre- post-, 172, USA]	Youth experiencing homelessness between the ages of 10 and 24 years, regardless of location or subgroup, whether living on the street or in service accommodations. This typically included more males than females. The proportion of youth with mental health or substance use issues varied greatly.	• Intensive case management • Independent living programs • Brief motivational intervention • Intensive case management, • cognitive behavioral intervention • living skills/vocational intervention • peer-based intervention • supportive housing.	The authors found that there was insufficient evidence to claim any clinical effectiveness in any of the interventions.
Coren 2016	Meta-analysis AMSTAR 14/16 Low quality review	“To evaluate and summarize the effectiveness of interventions for street-connected children and young people that aim to promote inclusion and reintegration, increase literacy and numeracy, facilitate access to education and employment, promote mental health, including self-esteem, reduce harms associated with early sexual activity and substance misuse”	n = 13 Baer (2007) [RCT, 127, USA] Carmona (2014)/Slesnick (2015) [RCT, 270, USA] Cauce (1994) [RCT, 115, USA] Hyun (2005) [RCT, 27, South Korea] Milburn (2012) [RCT, 151, USA] Nyamathi (2012/13) [RCT, 100,USA] Peterson (2006) [RCT, 285, USA] Rew (2007) [Quasi-RCT, 572, USA] Rotheram-Borus (2003) [CBA, USA, 311] Slesnick (2005) [RCT, 124, USA] Slesnick (2007/08) [RCT, 580, USA] Slesnick (2009) [RCT, 129, USA] Slesnick (2013)/Guo (2014) [RCT, 179, USA]	Street-connected children and young people between birth and 24 years of age regardless of location, reason for street connectedness or gender, including those living or working on the street or in public places, and returning to the family home at different times.	• harm-reduction • inclusion programs • reintegration programs • shelter • housing • drop in support • any type of intervention interventions explicitly aimed at reducing risky sexual activity and substance misuse • Individual • Family • Small groups • Entire communities. • Multi-faceted interventions that incorporate a range of approaches, including housing, education, training and health.”	There appeared to be no difference in effect between focused therapies and standard services for street-connected children and young people.
Noh 2018	Meta-analysis AMSTAR 8/13 Critically low quality review	“To examine the literature for psychological interventions directed toward runaway and homeless youth and to evaluate the effectiveness of these interventions in terms of mental health outcomes.”	n = 11 Baer (2007) [RCT, 127, USA] Brillantes-Evangelista (2013) [non-RCT, 29, Philippines] Hyun (2005) [RCT, 27, South Korea] McCay (2011) [non-RCT, 15, Canada] McCay (2015) [non-RCT, 89, Canada] Milburn (2012) [RCT, 151, USA] Peterson (2006) [RCT, 285, USA] Rew (2017) [non-RCT, 80, USA] Slesnick (2005) [RCT, 124, USA] Slesnick (2007) [RCT, 180, USA] Slesnick (2009) [RCT, 119, USA]	Youth experiencing housing instability that are 12–24 years of age. Most of the included studies included both males and females. Two of the studies included only females or males.	• Art therapy • Cognitive behavioral therapy (CBT) umbrella • Family therapy • Motivational interviewing • Strengths-based interventions	None of the psychological interventions appeared to have any effect on mental health outcomes. However, substance use appeared positively affected by Family Therapy, and depression appeared positively affected by CBT.
Xiang 2013	Systematic Review AMSTAR 7/13 Critically low quality review	“Primary: to summarize evidence on interventions for substance use among homeless youth Secondary: to draw implications for practice, to provide a critical appraisal of the methodologies in existing literature, and to suggest avenues for future research.”	n = 15 Peterson et al. (2006) [RCT, 185, USA] Baer et al. (2007) [RCT, 127, USA] Slesnick, Prestopnik, Meyers, et al. (2007) [RCT, 180, USA] Slesnick, Kang et al. (2008) [Longitudinal, 172, USA] Booth et al. (2008) [Crossover, 147, USA] Ferguson & Xie (2008) [Prospective, 28, USA] Cauce et al. (1994, 1998) [RCT, 304, USA] Souza et al. (2011) [Longitudinal, 400, Honduras] Stewart et al. (2009) [Prospective, 70, Canada] Slesnick, Bartle-Haring, et al. (2006)/Slesnick & Prestopnik (2005, 2009) [RCT, 243, USA] Milburn et al. (2012) [RCT, 151, USA] Steele & O’Keefe (2001) [Longitudinal, 106, USA] Rotheram-Borus (2003) [Prospective, 187, USA] Pollio et al. (2006) [Longitudinal, 371, USA] Kisely et al. (2008) [Retrospective, 45, Canada]	Youth experiencing homelessness between the ages of 12 and 24	• Brief motivational intervention • Community reinforcement approach • Knowledge and skills training • Case management • Peer support interventions • Family therapy • Shelter services • Supportive housing	Most studies showed improvements in substance use outcomes, however, improvements rarely varied between the treatment group and the control group. The only treatment shown to have greater relative efficacy was family therapy.

**Fig. 2:**
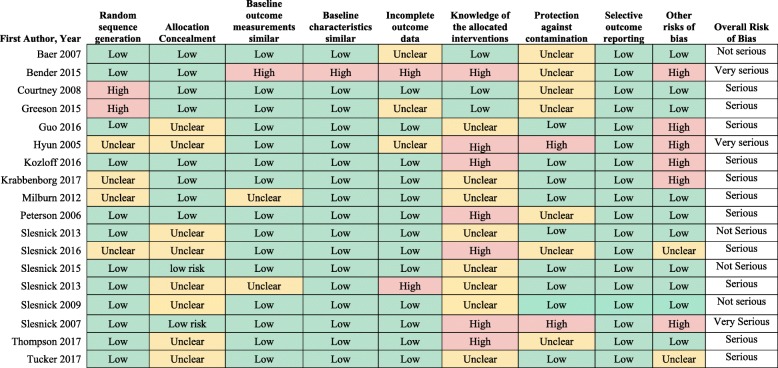
Methodological Quality of Included RCTs using Cochrane Risk of Bias Tool

Methodological quality of the included studies was low or very low, with serious risk of bias across most included studies (see Fig. 2 for RCTs and Table 4 for SRs). The most common domain with a high level of risk was knowledge of the allocated interventions, as blinding was often not possible or difficult with the nature of the interventions.

**Table 4:** Methodological Quality of Included Systematic Reviews using AMSTAR II

AMSTAR II Criteria	Quality Ratings for Systematic Reviews			
	Altena 2010	Coren 2016	Noh 2018	Xiang 2013
1. Did the research questions and inclusion criteria for the review include the components of PICO?	Yes	Yes	Yes	Yes
2. Did the report of the review contain an explicit statement that the review methods were established prior to the conduct of the review and did the report justify any significant deviations from the protocol? (critical)	No	Yes	No	No
3. Did the review authors explain their selection of the study designs for inclusion in the review?	No	No	Yes	Yes
4. Did the review authors use a comprehensive literature search strategy? (critical)	Partial yes	Yes	Partial yes	Partial yes
5. Did the review authors perform study selection in duplicate?	Yes	Yes	No	No
6. Did the review authors perform data extraction in duplicate?	No	Yes	No	No
7. Did the review authors provide a list of excluded studies and justify the exclusions? (critical)	No	Yes	No	No
8. Did the review authors describe the included studies in adequate detail?	Yes	Yes	Partial yes	Yes
9. Did the review authors use a satisfactory technique for assessing the risk of bias (RoB) in individual studies that were included in the review? (critical)	No	Yes	Yes	No
10. Did the review authors report on the sources of funding for the studies included in the review?	No	Yes	No	No
11. If meta-analysis was performed did the review authors use appropriate methods for statistical combination of results? (critical)	No meta-analysis was performed	Yes	Yes	No meta-analysis was performed
12. If meta-analysis was performed, did the review authors assess the potential impact of RoB in individual studies on the results of the meta-analysis or other evidence synthesis?	No meta-analysis was performed	Yes	No	No meta-analysis was performed
13. Did the review authors account for RoB in individual studies when interpreting/ discussing the results of the review? (critical)	No	Yes	Yes	Yes
14. Did the review authors provide a satisfactory explanation for, and discussion of, any heterogeneity observed in the results of the review?	No	Yes	Yes	Yes
15. If they performed quantitative synthesis did the review authors carry out an adequate investigation of publication bias (small study bias) and discuss its likely impact on the results of the review? (critical)	No meta-analysis was performed	No	No	No meta-analysis was performed
16. Did the review authors report any potential sources of conflict of interest, including any funding they received for conducting the review?	Yes	Yes	No	Yes
Overall Assessment of Quality	Critically low quality	Low quality	Critically low quality	Critically low quality

**Fig. 3:**
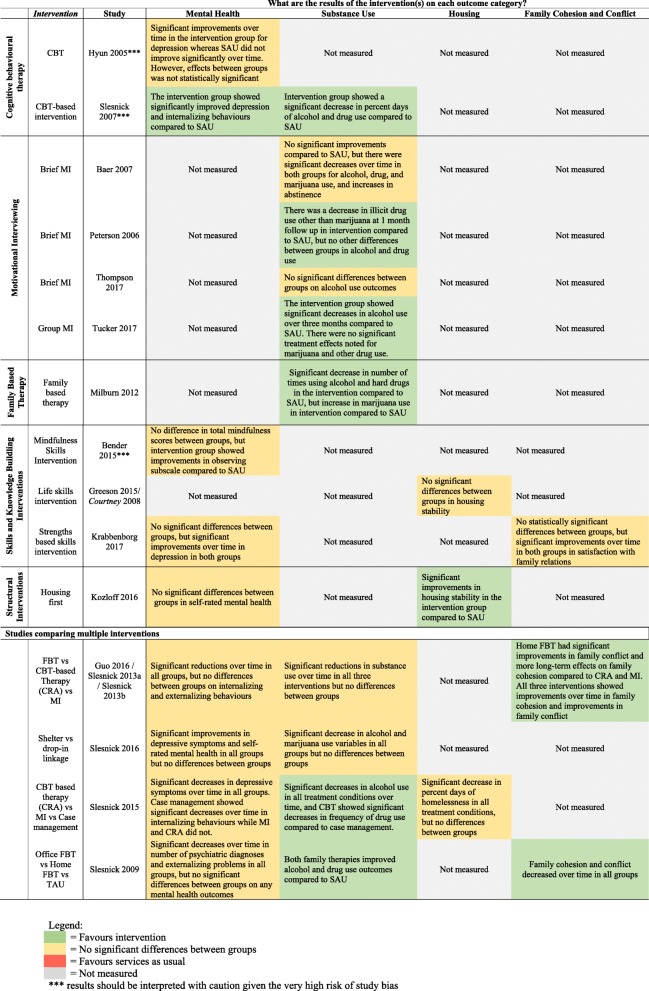
Visual Summary of Results of RCTs by Outcome

The main categories of interventions applied to youth homelessness included: 1) individual and family therapy (e.g. cognitive behavioural therapy (CBT), motivational interviewing (MI), family therapy), 2) skills building (e.g. life skills, mindfulness), 3) case management and 4) structural interventions (e.g. housing support, drop-in centres, shelters). See Table 5 for the definitions of interventions. The results of RCTs have been summarized using a visual map (see Fig. 3).

**Table 5:** Definitions of Interventions

Categories of Interventions	Intervention Type	Definition
1. Individual and family therapies	1a. Cognitive Behavioural Therapy (CBT)	A type of short-term psychotherapy, based on a pro-active and shared therapeutic relationship between a therapist and client, that enables an individual to develop skills and strategies to make sense of the present [19]. CBT is structured and time-limited (i.e. typically 6–20 sessions), and allows the client to identify, challenge and change thoughts, attitudes and beliefs that may trigger emotional and behavioural difficulties [46, 47, 49, 50]. Usually, CBT is effective in treating anxiety and depression, but also conditions such as bipolar disorder, schizophrenia and psychosis [19]. Includes: - *Community reinforcement approach* (CRA): a CBT-based therapy that recognizes the impact that the environment/community (i.e. family, hobbies, work, friends, etc.) can have on an individual. CRA permits the individual to modify environmental factors such as developing communication, problem solving and job skills, in order to support the recovery process [54]. - *Dialectical behaviour therapy*: the client is taught that their experiences and behaviours are valid (i.e. acceptance), and that, in order to move on and manage their emotions, they must make positive changes (i.e. change) [55].
	1b. Family Therapy	A type of psychotherapy that aims for family preservation by promoting support and understanding among family members during times of instability, uncertainty, anger, grief, or trauma [20, 29]. By providing a safe environment, Family Therapy focuses on intrapersonal factors that support family cohesion and re-establishing connections; it seeks to understand individual behaviour and interactions between the individual and their family in order to reduce defensive communication patterns. The duration of sessions is client-dependent, varying from a few sessions (2–3) to longer. Ecologically Based Family Therapy is a home-based model, while Functional Family Therapy is provided in a professional setting [46, 47, 49].
	1c. Motivational Interviewing	A collaborative, person-centered counselling approach based on empathy and self-efficacy that is often used to address risky sexual health behaviours, alcohol and drug use, and mental health issues [21, 48]. Motivational interviewing can be a single session or multiple sessions with a clinical psychologist or other trained health workers, with the objective of building self-confidence and developing independence to strengthen the motivation for change [56].
2. Skill building programs	Life skills training program Mindfulness Strengths-based	Life Skills Training enables youth 16 years and older to adopt and develop key competency skill areas in education, employment, daily living skills, survival skills, choices and consequences, and interpersonal/social domains. Life Skills Training also includes an extensive outreach component in order to recruit youth into the program and provide short-term case management support [40, 41]. Mindfulness (SAFE intervention): Through a three-day workshop, youth are invited to adapt concepts of mindfulness, with a focus on internal, interpersonal, and environmental cues, and fostering assertiveness and problem- solving skills, and strategies for asking for help [42]. Strengths-based intervention (Houvast) enables and promotes self-agency in his or her own recovery process, by goal-setting, identifying ineffective strategies and problems in the way of achieving set goals [39].
3. Case management		Case management is health and social service where an individual is assigned a case manager who plans and facilitates access to health and social care services required for recovery [22]. Intensive case management is provided to individuals with serious mental health disorders and struggling with addictions [57]. The case manager accompanies the service user to meetings and can be available for up to12 hours per day, 7 days a week. One form of time-limited intensive case management is critical time intervention, which supports continuity of care and facilitates access to services for clients during transitions (e.g. from a shelter to independent housing or following discharge from a hospital) [43]. Critical time intervention is often offered for a period of 6–9 months.
4. Structural Support	4a. Housing Programs	Housing First is a housing model that provides immediate access to permanent independent housing in the community and is not contingent on sobriety or abstinence or treatment. Individuals enrolled in the Housing First program are typically given access to scattered-site housing of their choice with mobile and off-site mental health services. Supported Housing: safe and affordable housing with integrated health and social support services [35]. The supportive service (usually Assertive community treatment) is provided by a multidisciplinary team. Independent Living Programs aim is to provide homeless and vulnerably housed youth with life skills through a structured and supervised residential [33].
	4b. Drop-in Centre 4b. Shelter Services	Drop-in Centers: offered for youth 24 h/7 days a week, and provides access to food, laundry, and shower facilities, as well as recreational activities (e.g. television, books, board games or video games), and opportunities for socialization [44]. Drop-in staff often link youth with community resources (i.e. counseling and housing programs). Shelter Services: provide a temporary overnight alternative to street living, and is open 24 h/day, 7 days a week [44].

### Individual and family therapy

#### Cognitive Behavioural therapy

CBT led to improvements in substance use and depression, and one systematic review also reported improvements in internalizing behaviours and self-efficacy [33–36]. When a CBT-based therapy (community reinforcement approach) was delivered with case management in one study, there were improvements in percentage of days being housed, psychological distress, and substance use [33]. Two systematic reviews conducted meta-analyses on CBT and CBT-based interventions and found no statistically significant difference in mental health outcomes compared to services as usual, but noted that lack of a statistically significant difference may be due to heterogeneity between studies [34–36].

#### Family therapy

Family-based therapy was delivered in an office setting, known as functional family therapy, or in the home setting, called ecologically-based family therapy. Systematic reviews reported that all three family therapy RCTs showed a reduction in substance use [34–36]. However, Noh (2018) conducted a subgroup meta-analysis on two family intervention studies and found no significant effect on substance use [34]. Another meta-analysis found a statistically significant improvement in family cohesion, but called it a clinically marginal effect [36]. In a three arm RCT comparing home-based family therapy with MI and a CBT-based therapy, all three groups improved over time in internalizing and externalizing behaviours, family cohesion, and substance use [47–49]. Furthermore, when an RCT compared functional family therapy, home-based family therapy, and services as usual, all treatments showed improvements in days living at home at three, nine and 15 months, but no group was superior to another [52].

#### Motivational interviewing

Brief or group MI interventions were primarily designed to address substance use and/or risky sexual behaviours. A brief intervention showed declines in non-marijuana drug use at 1-month follow up, but the reduction was no longer significant after 3 months [33–35]. In another RCT, both the service as usual and intervention groups showed significant improvements over time, but there were no significant and durable results in favour of the experimental group [21]. A 16-week group MI intervention found significant declines in alcohol use and increased motivation to change drug use, but no significant decreases in marijuana use [37]. A two-session individual brief MI intervention compared to an education program reported significant improvements in readiness to change alcohol use [38].

### Skill building

The interventions focused on vocational and life skills, mindfulness, and strengths-based skill building. One systematic review included one study evaluating a life skills intervention and found improvements in family contact and near significant improvements in depressive symptoms [33]. Another systematic review reported similar results but noted an increase in substance use over 6 months which could not be explained [35]. A training program based on a peer influence model showed non-statistically significant decreases in drug use in the treatment group. One study evaluated a strengths-based program deployed in a shelter to identify and make use of strengths in each youth [39]. This program showed no significant differences between groups but found improvements over time in depression, substance use, and satisfaction with family relations [39]. Two RCTs evaluated a vocational and life skills program and a mindfulness skills program, though did not report promising treatment effects [40–42].

**Fig. 4:**
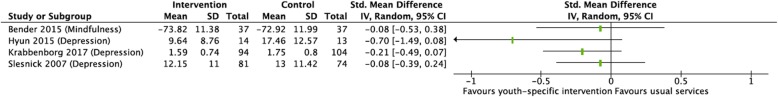
Intervention vs. Usual services for Short Term (0-6 months) Mental Health Outcomes)

We attempted to conduct meta-analyses whenever possible, but due to the heterogeneity between studies, it was inappropriate to pool the results into a combined effect size. As such, we developed a forest plot for short-term mental health outcomes of a mindfulness intervention, CBT intervention, strengths-based intervention, and CBT-based intervention [39, 42, 50–53]. The figure depicts a general trend favouring the interventions but none reaching statistical significance compared to control (see Fig. 4).

### Case management

Two systematic reviews reported on several case management programs, including intensive case management and multidisciplinary case management, and reported minimal additional benefit of the programs relative to their comparison interventions [33–35]. They noted that one program showed favourable results for substance use, but the study quality was very low due to low retention rates [33]. In a three-arm RCT, case management, a CBT-based intervention, and MI all showed significant improvements over time in housing stability, depression, and substance use, but no significant differences between groups [45]. Case management led to improvements over time in internalizing behaviours while the other groups did not [45]. Overall, there is evidence to suggest that case management may have impacts on substance use, depression, and housing stability, but different control conditions in each of the studies made it difficult to assess overall effectiveness of the intervention.

### Structural support

#### Housing programs

A subgroup analysis of young adults in an RCT of the housing first model for adults with mental illness found that, compared to treatment as usual, housing first significantly increased the proportion of days stably housed over the 24-month trial, but had no impact on self-rated mental health [43]. One systematic review included an independent living program and reported marginal results on psychological measures, however reported some positive outcomes on housing status [33]. The same systematic review also included a study evaluating a supportive housing program, which reported lower rates of substance abuse and improvements in self-reported health, but the study quality was noted to be low. Xiang evaluated the same supportive housing program and also concluded that the lower rates of substance use may be attributed to baseline differences between control and intervention groups instead of treatment effect [35].

#### Drop-in and shelter services

A systematic review included three shelter services studies, two evaluating residential services and one evaluating emergency shelter and crisis services [35]. The review showed some improvements in substance use but this was not consistent over the various studies and there were no enduring effects over time. An RCT compared referrals from case management made to drop-in versus shelter services programs [44]. There were no differential treatment effects, as both groups showed decreases in depression and substance use over time [44]. However, individuals assigned to the drop-in service had greater service contacts and access to care over 6 months [44].

### Gender and equity analysis

Equity variables were not consistently measured, reported, or analyzed across studies. Several studies measured equity and PROGRESS+ factors with baseline sample characteristics, but very few included them as covariates. The most examined factors were gender and ethnicity/race, with some studies mentioning place of residence and occupation. A number of RCTs included equity variables in their analysis [21, 37, 39–41, 43–49], as did three systematic reviews [34–36].

A number of studies indicated that females responded differently to services than males. Slesnick’s studies have showed that females initially reported higher rates of depression than males, with a greater reduction throughout the study [44–46]. Female adolescents showed a greater improvement in family cohesion subsequent to treatment regardless of the treatment condition [47] and appeared to derive greater benefit from shelter services than males [35].

Some variance in relation to ethnicity and employment emerged as well. While youth from ethnic minorities had greater reductions in substance use, they also relapsed more quickly than white youth [49] and had more HIV risk behaviours [44]. African Americans showed a greater reduction in percent days homeless than other ethnic groups [45]. Non-Hispanic white youth more quickly reduced their number of days drinking to intoxication [44]. Those employed or in school at baseline were more likely to remain employed at follow-up [39].

## Discussion

This review identified a wide variety of interventions for youth experiencing housing instability. Regarding individual and family therapies, CBT interventions showed improvements in depression and substance use outcomes [33–36]. Family interventions led to improvements in alcohol and drug use measures and may have had an impact on family cohesion [34–36]. Motivational interviewing, skill-building programs and case management showed inconsistent effects on mental health and substance use when compared with services as usual and other interventions [21, 33, 35–42, 45–49]. Among the structural support interventions, housing first led to improved housing stability outcomes, while drop-in and shelter services led to inconsistent effects [43, 44]. The equity analysis revealed differential treatment effects based upon gender and ethnicity, with females often deriving more treatment benefit than males [44, 45, 47–49]. Equity analyses were limited, with very little mention of important considerations such as sexual orientation status, as LGBTQ+ youth are disproportionately represented in the homeless population [58, 59].

While in many circumstances, differences were not statistically significant between treatment groups, this does not preclude the lack of effectiveness of these interventions. It is important to note that a treatment as usual group was not the absence of an intervention, but rather involved referral to other community services and follow-up with researchers. This may lessen the differences between the intervention and control arms, and decrease the detectable effect of the intervention. Providing non-specific support for youth may be enough to improve outcomes and reduce the toxic effects of adverse childhood experiences. However, that regression to the mean may also potentially explain the changes observed over time [60]. As participants may enter the research studies during a point of crisis, they may naturally improve over time regardless of the study group, and this effect may lessen the observed differences between intervention and control groups.

### Tailoring interventions to the needs of youth

The dynamics of youth homelessness are complex; pathways to housing are precarious, sociocultural backgrounds are becoming increasingly diverse and available resources are inconsistent. Research has shown that unstable family relationships underlie youth homelessness, and many youth have left homes where they experienced interpersonal violence and abuse [3–5, 61]. Among these difficult family issues, other personal factors arise as a result of their environmental contexts, which can interplay and lead to increased distress. These challenges include substance use, depression, and disability, and can compoundly contribute to strain [10]. The interventions identified in this review may help to address the specific needs of youth and may be tailored to their situation.

One important consideration to note is that while we have defined youth as those ages 13 to 24 for the purposes of this study, this grouping brings together minors as well as young adults of legal age. While this age categorization is reflective of the literature on the youth population, we recognize that there are differences between the experiences of younger versus older youth. Furthermore, there are medicolegal implications of the mature minor and capacity to consent. Clinicians and program implementers who work directly with this population need to consider the ethical considerations of consent for treatment participation with mature minors as well as the legal obligations provided by their governing college [62].

### Strengths and limitations of the review

We conducted a high quality search, complying to PRISMA-E guidelines [26]. This review included only high quality study designs: RCTs and systematic reviews. This may, however, have limited the types of interventions that were included. Limitations include a broad range of outcomes and, thus, too few studies available for meta-analyses. There was heterogeneity in the interventions, and the available evidence was insufficient to use network meta-analysis to answer the question of the relative advantages of the different types of interventions. In our systematic review, the studies did not use placebo designs and, instead, used several different interventions/comparisons. However, there was considerable heterogeneity in the outcome measures and this prevented a pooling of the effects. The services-as-usual comparisons were often not adequately described in the primary studies, limiting the comparisons that could be made across different studies. Furthermore, our definition of youth experiencing homelessness focused on unaccompanied youth and did not include accompanied youth that enter homeless situations along with their families, as this youth population has quite distinct circumstances and needs.

### Implications for future research, policy, and practice

The results suggest that tailored interventions for youth may have impacts on depression, substance use and housing. Given the diverse pathways to youth homelessness, health care policy-makers, practitioners and other stakeholders should consider the specific needs of youth during prevention and delivery of care. Furthermore, we recommend additional high quality research to be conducted in the area of family-based therapies, CBT, and housing interventions, which have shown some positive results thus far. We further recommend additional considerations for equity factors. Few studies examined equity factors, and those that did were limited largely to gender and ethnicity. There remains a large gap in data regarding the intersectionality between a variety of PROGRESS+ factors contributing to youth experiences.

There is also a large gap in research on the impact of structural interventions such as housing and case management on youth experiencing homelessness. The predominance of psychological and family interventions in this paper suggests that more work could be done to study an area in which it may be more difficult to design studies. Nonetheless, future research on these interventions are important to addressing the root causes of poverty and homelessness. Furthermore, there are emerging models of housing which have not yet been evaluated rigorously in the literature. For instance, host homes provide safe and temporary housing for up to 6 months for youth while supporting them with a case manager to identify long term solutions [63]. Rapid re-housing programs provide short-term subsidies to allow persons experiencing homelessness to acquire stable housing as quickly as possible [64, 65]. The landscape on housing models continues to evolve and future research will need to evaluate these in the context of youth experiencing homelessness.

## Conclusion

This review identifies a variety of interventions targeted towards the unique needs of youth experiencing homelessness. CBT interventions may lead to improvements in depression and substance use, and family-based therapy may impact substance use and family outcomes. Housing programs may lead to improvements in housing support and stability. Other interventions such as skill building, case management, show inconsistent results on health and social outcomes.

## Supplementary information

**Additional file 1.** Search Strategy.

**Additional file 2.** Interventions for Social, Personal, Health and Social Service Utilization, and Sexual Health Outcomes.

**Additional file 3.** PRISMA Equity Checklist.

## Data Availability

Not applicable.

## Abbreviations

AMSTAR II: A MeaSurement Tool to Assess systematic Reviews
CBT: Cognitive Behavioural Therapy
MI: Motivational Interviewing
PRISMA-E: Preferred Reporting Items for Equity-Focused Systematic Reviews and Meta-analyses
PROGRESS+: Place of Residence- Race/ethnicity/culture/language- Occupation- Gender/sex- Religion- Education- Socioeconomic status- Social capital **+** refers to: 1) personal characteristics associated with discrimination (e.g. age, disability). 2) features of relationships (e.g. smoking parents, excluded from school). 3) time-dependent relationships (e.g. leaving the hospital, respite care, other instances where a person may be temporarily at a disadvantage)
RCT: Randomized Control Trial
