# Supplementary material for: The impact of interventions for youth experiencing homelessness on housing, mental health, substance use, and family cohesion: a systematic review
Source: BMC Public Health. 2019 Nov 14;19:1528. doi: 10.1186/s12889-019-7856-0 (PMC6857126; doi:10.1186/s12889-019-7856-0)
Supplement: Supplementary file 1 — Additional file 1. Search Strategy. [file 12889_2019_7856_MOESM1_ESM.docx]

**Additional file 1. Search Strategy**

| 1 vulnerable populations/  poverty areas/  2 ((deprived or destitute? or impoverished or low income or marginalised or marginalized or needy or poverty or vulnerable) adj2 (adolesc$ or child$ or famil$ or men or people or youth? or women)).tw,kf.  3 homeless persons/ homeless youth/ runaway behavior/  4 (homeless$ or runaway?).tw,kf.  5 (temporar$ adj2 (accommodat$ or home? or hous$)).tw,kf.  6 ((based or housed or residen$ or temporar$) adj2 shelter?).tw,kf.  7 or/1-7  8 exp program evaluation/  9      (effectiveness or initiative? or prevent$ or program$ or reduc$ or strateg$ or treatment).tw.  10 or/8-9  11      systematic review/ meta analysis/ randomized controlled trial/ controlled clinical trial/ pragmatic clinical trial/ controlled before-after studies/ interrupted time series analysis/ controlled before-after studies/ (randomised or randomized).ab,kf.  12 (before adj2 after adj5 (design$ or study or trial)).tw,kf.  13      ((preintervention? or pre intervention? or postintervention? or post intervention?) adj5 (study or trial)).tw,kf.  14 ((pre test or pretest or (posttest or post test)) adj2 (design$ or method$ or study or trial)).tw,kf.  15      *economics/ exp *"Costs and Cost Analysis"/ economics, nursing/ economics, medical/ economics, pharmaceutical/ exp economics, hospital/ economics, dental/ exp "Fees and Charges"/ exp budgets/  16 ((budget$ or economic$ or cost or costs or costly or costing or price or prices or pricing or pharmacoeconomic$ or pharmaco-economic$ or expenditure or expenditures or expense or expenses or financial or finance or finances or financed) adj6 (analys$ or analyz$ or effect$ or evaluat$ or impact$)).ab. /freq=2  17 (cost$ adj2 (effective$ or utilit$ or benefit$ or minimi$ or analy$ or outcome or outcomes)).ab,kf.  18 (value adj2 (money or monetary)).tw,kf.  19 exp models, economic/ economic model$.ab,kf. |
| --- |
