# Supplementary material for: The impact of interventions for youth experiencing homelessness on housing, mental health, substance use, and family cohesion: a systematic review
Source: BMC Public Health. 2019 Nov 14;19:1528. doi: 10.1186/s12889-019-7856-0 (PMC6857126; doi:10.1186/s12889-019-7856-0)
Supplement: Supplementary file 2 — Additional file 2. Interventions for Social, Personal, Health and Social Service Utilization, and Sexual Health Outcomes. [file 12889_2019_7856_MOESM2_ESM.docx]

**Additional file 2. Interventions for Social, Personal, Health and Social Service Utilization, and Sexual Health Outcomes**

*Interventions for Social and Personal Outcomes*

This category reflects important outcomes regarding the quality of life, social support and stability, self-esteem, self-efficacy, education and employment of youth experiencing homelessness. In terms of interventions that showed some positive results, a CBT intervention showed significant improvements in self-efficacy over time, but not self-esteem, and community reinforcement approach significantly improved social stability compared to treatment as usual (Hyun, Chung & Lee, 2005; Slesnick et al, 2007). A housing first program significantly improved condition-specific quality of life at 6 months, and the QOLI-20 subscale of leisure at 6, 12, 24 months compared to usual care, but the program was associated with lower rates of employment over the two-year study period (Kozloff et al, 2016).

Other interventions showed mixed results on social and personal outcomes. Krabbenborg et al (2017) evaluated a strengths-based intervention which showed improvements over time in terms of quality of life, satisfaction with finances, satisfaction with health, care needs, autonomy, competence, resilience, and employment/education rates, but no more than treatment as usual. The intervention group also showed a decline over time on satisfaction with social relations. A Life Skills Training program found no impacts on economic well-being, income and net worth, education, employment, sense of preparedness, financial accounts, personal documentation, and social support outcomes (US Department of Health and Human Services, 2008; Greeson, 2015).

*Interventions to Reduce Violence*

Violence outcomes were measured as the likelihood of victimization (Slesnick et al, 2015; Petering, Wenzel, & Winetrobe, 2014), delinquency (Milburn et al, 2012), and aggression (Coren et al, 2016). Positive results were found for a family intervention, which showed a reduction in delinquent behaviours (Milburn et al, 2012).

Other results were varied. Slesnick et al (2015) showed that case management and CBT-based therapy resulted in reductions in victimization, but that motivational enhancement technique did not improve this outcome. Coren et al (2015) reported an overall reduction in verbal aggression and family violence, but noted that there were no significant differences for interventions to reduce harmful behaviour and lifestyles for homeless youth. A life skills training program showed no improvement on delinquent behaviours (Courtney et al, 2008).

*Interventions for Health and Social Service Utilization*

One study measured health service utilization using the number of visits in the prior 30 days to the drop-in center, the number of utilizations of additional services (e.g. case management, recreation, life discovery classes and spiritual activities) offered by the agency (Baer et al, 2007). This study found that brief motivational intervention had an overall decrease in health service utilization. Slesnick and colleagues (2016) measured the frequency of contacting various services (i.e. housing services, legal issues, shelter), and while shelter and drop-in linkage both decreased the number of service contacts in the past 30 days, the difference was not significant. A housing first program showed no significant differences in use of services from medical, clinical and social providers as well as emergency department visits (Kozloff et al, 2016).

*Interventions for Sexual Health*

Sexual health outcomes were found in five studies (Milburn 2012; Tucker 2017; Thompson 2017; Slesnick 2016; US Department of Health and Human Services, 2008) and one systematic review (Coren 2016). The measured outcomes related to sexual health education, sexual risk-taking behaviors, HIV and/or sexual health service utilization, and pregnancy. In the systematic review, Coren (2016) examined five studies evaluating whether interventions caused a reduction in harms associated with early sexual activity, such as number of partners, HIV knowledge, unprotected sex. The authors concluded that although some studies showed some benefit, the results were mixed and there were overall no consistent intervention effects.

Regarding the RCTs, a study evaluating a motivational interviewing intervention found no significant effect on sexual activity/frequency, willingness to change behaviors, condom usage, and/or condom use self-efficacy (Tucker et al, 2017). Similarly, Thompson et al (2017) evaluated a brief intervention compared to an education comparison for reducing homeless youth’s sexual risk behaviors. While the brief intervention was not found to have any significant effect, they did find that the educational comparison reduced the number of times youth engaged in unprotected sex, and increased their readiness to change HIV sexual risk behavior. Slesnick et al (2016) found youth in both drop-in and shelter linkage conditions showed statistically significant increases in service use including HIV/STI related services. Youth in the drop-in linkage condition were found to report a greater increase in HIV knowledge than youth in the shelter linkage condition. A RCT evaluating a knowledge and skills development program, the Life Skills Program, found no statistically significant differences between intervention and treatment as usual on pregnancy rates over a two-year follow-up period (Courtney et al, 2008).
